# Improving the Dehydroepiandrosterone Induced PCOS Rat Model: Interplay of Age, High Fat Diet, and Treatment Regimen on Reproductive and Metabolic Phenotypes

**DOI:** 10.1007/s43032-024-01742-1

**Published:** 2024-11-20

**Authors:** Pooja Mallya, Guruprasad Kalthur, Anne Boyina Sravani, Shaila A. Lewis

**Affiliations:** 1 Department of Pharmaceutics, Manipal College of Pharmaceutical Sciences, Manipal Academy of Higher Education, Manipal, 576104 Karnataka India; 2 Division of Reproductive Biology, Department of Reproductive Science, Kasturba Medical College, Manipal Academy of Higher Education, Manipal, 576104 Karnataka India

**Keywords:** Animal model, DHEA, High fat diet, Phenotype, Polycystic ovary syndrome

## Abstract

Polycystic ovary syndrome (PCOS) is a ubiquitous reproductive condition with triggering hallmarks such as glucose intolerance, hyperandrogenism, and dyslipidemia. Despite the existence of various PCOS animal models, an ideal model which could encompass all PCOS-specific phenotype is of dire need. Dehydroepiandrosterone (DHEA) induced PCOS rats are frequently employed; though, determining the superior model among pubertal and prepubertal rats, incorporation of high fat diet (HFD), and their sustainability remains uncertain. This study aims to examine the age factor, impact of HFD, and DHEA regimen in model development. Prepubertal and pubertal Sprague–Dawley rats were subcutaneously injected with DHEA (6 mg/kg and 60 mg/kg/day, respectively) with and without HFD up to 21 days. Serum testosterone, glucose, lipid profile, ovary morphology, and estrous cycle were evaluated. Following 21 days of treatment with DHEA, pubertal PCOS rats exhibited better reproductive phenotype than prepubertal rats. However, there was no significant difference in the lipid profile. Accordingly, both the age-group rats were concomitantly treated with DHEA and HFD for additional 3 weeks on alternate day basis after model development. The persistence of reproductive and metabolic features on treatment withdrawal were also simultaneously investigated by alienating the rats into continuous and stop dosing groups. The DHEA + HFD and DHEA treated pubertal rats in continuous dosing group showed significant PCOS features (*p* < 0.05) compared to stop dosing, prepubertal, and control groups. To conclude, continual dosing with DHEA on alternate days for 3 weeks is necessary to sustain metabolic and reproductive phenotypes of PCOS.

## Introduction

Polycystic ovary syndrome or PCOS affects reproductive age females with potential jeopardy of infertility. PCOS is a multifaceted condition affecting endocrine, metabolic, reproductive, and psychological functions in women. Worldwide, 2 to 26% women suffer from PCOS, nevertheless 70% women remain undiagnosed [1]. Women with PCOS display dyslipidaemia, elevated testosterone level, and impaired level of fasting glucose [2]. Androgen excess in ovaries is a major hallmark of PCOS [3].

Animal models play an imperative part in elucidating underlying mechanisms and pathogenesis of PCOS. The Rotterdam consensus criteria for PCOS diagnosis (2003) entails the presence of two out of three specific features: hyperandrogenism, polycystic ovaries, or oligo- or anovulation. An animal model exhibiting a single feature of these criteria cannot be deemed PCOS-like per se. A PCOS animal model must manifest at least two of the Rotterdam diagnostic criteria of PCOS to be considered equivalent to PCOS in women [4]. To date, an animal model exhibiting all the PCOS phenotypes is not yet established. While some models display only reproductive phenotypes, others display metabolic features [5].

Estrogen induced PCOS models disrupt estrous cycle regularity while suppressing production of androgen. Moreover, preclinical studies using estrogen model of PCOS often overlook the androgen level measurement leading to indecisive outcomes [6]. Although the Letrozole model is widely used, it fails to display metabolic features of PCOS. Dihydrotestosterone prepubertal model display irregular estrous cycle and reduced glucose tolerance, but maintain normal serum estrogen, testosterone, body weight, insulin levels, and insulin sensitivity which fails to mimic human PCOS phenotype [7].

The DHEA induced PCOS rat model is extensively used since it is the first androgen to appear in adolescent years of women and 25% women with PCOS report elevated DHEA level [8]. DHEA, a precursor to testosterone is a main circulating steroid hormone produced in the adrenal cortex and ovaries. It is known to regulate hypothalamus-pituitary-ovary axis function [9]. The rats injected with DHEA stimulates oxidative stress and inflammation resulting in the formation of large follicular cysts in ovaries. Additionally, it results in excessive androgen, testosterone, anovulation, insulin resistance, glucose intolerance, which mimic PCOS features in women [10, 11]. The discrepancy in reproducing metabolic phenotypes limits its reliability and reproducibility [12].

Kim et al. (2018) reported post pubertal rats treated with DHEA to be an ideal PCOS model compared to prepubertal PCOS rat model. Exposure to DHEA at prepubertal stage intruded on sexual maturity, induced labial fusion, presented normal level of serum testosterone, low ratio in polycystic ovaries and estrous cycle irregularity. On the contrary, exposure of rats to DHEA at or after attaining puberty resulted in anovulation, uterine dysfunction, increased polycystic ovary morphology thereby produced more stable model of PCOS. Nevertheless, there was no mention on metabolic phenotype [8]. Owing to the positive correlation of endocrine and metabolic disorders in PCOS, androgen treatment combined with high fat diet (HFD) is reliably used to develop an animal model of PCOS [13, 14]. Wu et al. (2023) reported the need for consecutive treatment with DHEA with or without HFD for 2 additional weeks even after 21 days of induction to sustain PCOS phenotypes in mice and initiate therapeutic interventions. Nevertheless, the study did not report changes in the lipid profile and glucose levels [15]. There is a need to study the impact of DHEA and HFD in model development based on age of rats and to validate the sustainability of model for long-term medical interventions. Therefore, we addressed the aforementioned limitations of pubertal animal model by treating the rats with DHEA with and without HFD for 21 days and consecutive treatment for two weeks to monitor serum testosterone levels, blood glucose, serum lipid profile, estrous cycle, and polycystic ovary morphology.

## Materials and Methods

### Materials

Pubertal (200-250 g; 45 days old) and prepubertal female Sprague–Dawley rats (~ 150 g, 21 days old) were procured from the Central Animal Research Facility, Manipal. The rats were kept in sterile condition with 12 h each of dark and light cycle, ambient temperature with food and water access. The study approval was obtained from Institutional Animal Care and Use Committee, Manipal Academy of Higher Education (IAEC/KMC/45/2022). DHEA was procured from TCI, India and the HFD was procured from the Star Enterprise, Mumbai, India.

### Experimental Design of Animal Study

Female Sprague Dawley rats (pubertal: 45 days old; 200-250 g body weight and prepubertal: 21 days old; ≈150 g body weight) were randomly divided into 17 groups, with six rats in each group. The grouping was done to select the finest model among pubertal and prepubertal rats, to verify the inclusion or exclusion of HFD with DHEA, and also to evaluate the persistence of the selected DHEA model. The groups were as follows, the control groups of pubertal and prepubertal rats that received no treatment, a high fat diet group, PCOS induced groups including prepubertal rats treated with DHEA and DHEA + HFD, pubertal rats treated with DHEA and DHEA + HFD, control and PCOS groups with pubertal and prepubertal rats consecutively treated with DHEA and DHEA + HFD for additional 21 days. The PCOS group containing pubertal rats received subcutaneous injection of DHEA (60 mg/kg/day) dissolved in 0.2 ml sesame oil, and the prepubertal rats in treatment group received 6 mg/kg DHEA daily for 21 days. The rats in continue dosing group received the same treatment for additional 21 days on alternative day basis.

### Induction of PCOS

Prepubertal and pubertal rats in PCOS group were administered subcutaneous injection of DHEA (6 mg/kg and 60 mg/kg, respectively) in 0.2 ml sesame oil daily up to 21 days. Following induction, blood was withdrawn by retro-orbital bleeding method, serum was collected, and analysed for lipid profile, testosterone, and glucose levels [8].

### Body Weight Measurement

The rats’ body weight was documented weekly throughout the length of experiment.

### Evaluation of Estrous Cycle

Visual assessment and vaginal cytology are the widely used methods to monitor estrous cycle in rats, the latter being more accurate [16]. In the current study, vaginal cytology was followed where a vaginal smear test was done in each rat prior to the end of induction period. Approximately 0.2 ml saline was flushed into the rat vagina by gently inserting a microtip attached to pipette at a depth of 5 to 10 mm into the vaginal orifice. Following the lavage, a tiny drop of sample was mounted on a slide, enclosed with cover slip, and then observed under microscope at a magnification of 45 × to determine the predominant type of cell in the epithelium of vagina [17].

### Biochemical Examination

Following the induction, blood glucose level, testosterone level, and lipid profile were measured. Serum testosterone assay and lipid profile test to measure total cholesterol (TC), triglycerides (TG), low-density lipoprotein (LDL-C), and high-density lipoprotein (HDL-C) serum levels were done by commercial assay tools especially chemiluminescence-immunoassay [18, 19]. Fasting blood glucose level was measured by glucose meter (Accu-Chek Active glucose meter).

### Histopathology Evaluation

After 21 days, the rats were euthanized humanely by cervical dislocation and their ovaries were excised. The tissue was kept in 10% formalin for 48 h and then processed. The tissue was transferred from wax bath and embedded into a molten paraffin wax mold. Using a microtome, three thin sections approximately 4µ of tissue block were longitudinally cut. In a water bath maintained at 50—52 °C, the sections were floated, mounted on microscopic slides, and stained by means of Haematoxylin and eosin (H & E). After placing the coverslip, the obtained slides were seen under the LX-500 LED trinocular Research microscope (Labomed) and images were taken with MiaCam CMOS-AR 6-pro microscopic camera allied with AR pro imaging software. The corpus luteum and various phases of follicles were manually counted in the tissue sections [20].

### Statistical Analysis

The body weight, blood glucose, serum testosterone assay, and lipid profile data are presented as mean ± SD. GraphPad Prism 9.2 software was used to perform statistical analysis. Effect of the treatment following 21 days of induction was analysed by one-way analysis of variance (ANOVA) followed by Bonferroni multiple comparisons test and the P-value < 0.05 was considered significant. The effect of continuous treatment with DHEA for 3 weeks post induction of PCOS was analysed by two-way ANOVA coupled with Tukey’s multiple comparison test. *P* < 0.0001 was regarded statistically significant.

## Results

### Body Weight, Blood Glucose, Serum Testosterone, Lipid Profile, Estrous Cycle, and Ovarian Morphology After 21 Days of PCOS Induction

**Fig. 1:**
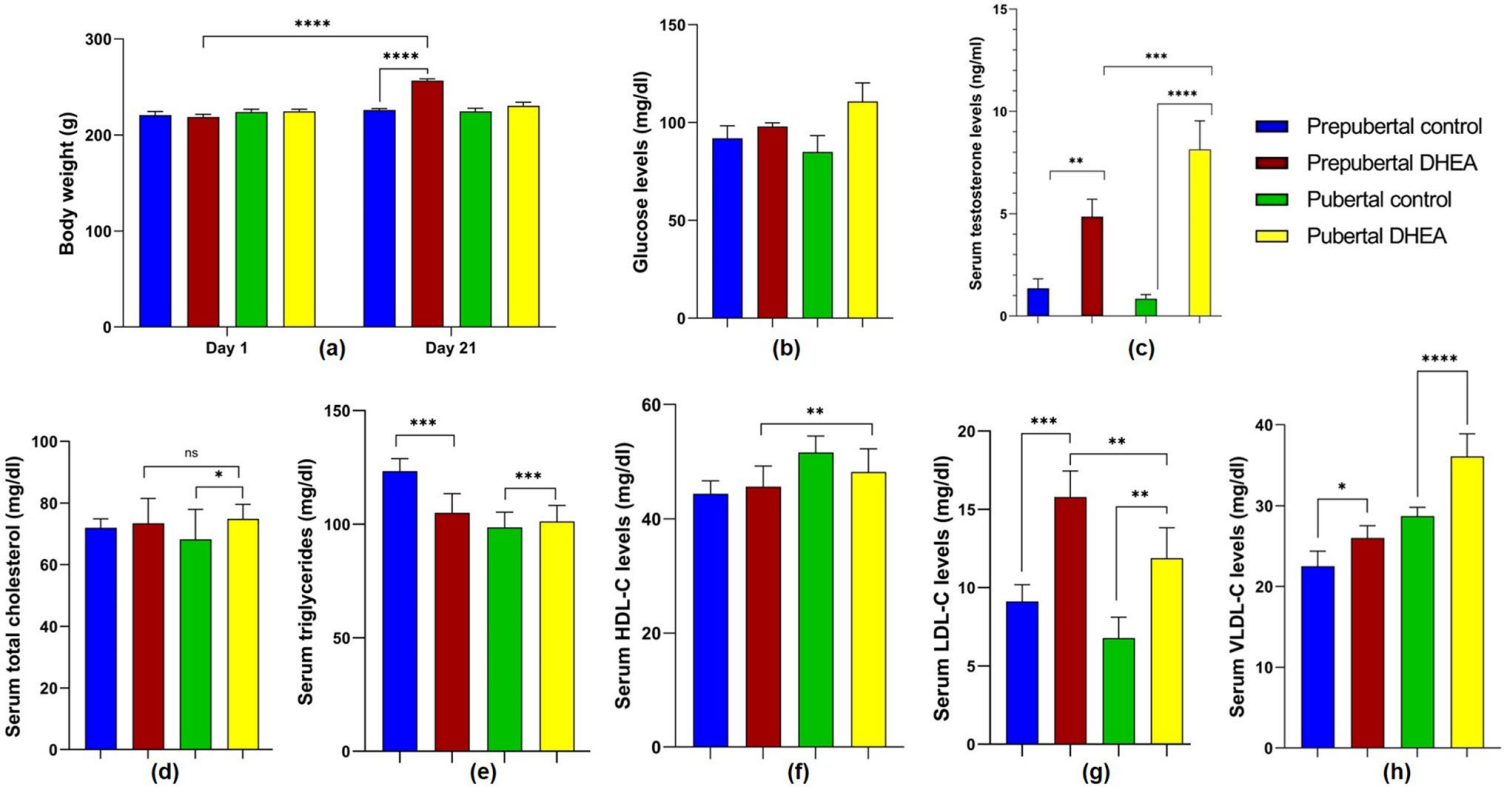
**a** Body weight, **b** blood glucose, **c** serum testosterone, **d** serum total cholesterol, **e** serum triglycerides, **f** serum HDL, **g** serum LDL, and **h** serum VLDL levels in DHEA treated and control groups of female Sprague Dawley rats. The data are presented as mean ± SD and analysed by one-way ANOVA with Bonferroni’s multiple comparison test, except for the body weight analysed by two-way ANOVA using Tukey’s multiple comparison test. (ns indicates not significant, **, ***, **** denotes levels of significance)

The body weight of rats was observed weekly for 3 weeks. The prepubertal rats treated with DHEA showed significant increase (*P* < 0.05) in body weight after 21 days compared to pubertal rats treated with DHEA and the control group (Fig. 1a). Pubertal DHEA treated rats showed significant increase in blood glucose compared to prepubertal DHEA treated rats (*P* < 0.0001) (Fig. 1b). Serum testosterone levels were significantly higher in pubertal DHEA rats (*P* < 0.0001) compared to prepubertal DHEA and control group (Fig. 1c). The lipid profile test revealed that the PCOS groups had normal serum TC concentrations (P > 0.05) compared to control (Fig. 1d). The serum TG levels were significantly lower (*P* < 0.0001) in prepubertal DHEA treated rats compared to prepubertal control (Fig. 1e). Whereas, the pubertal PCOS group exhibited increased serum TG concentration than pubertal control (*P* < 0.0001). The serum HDL levels were significantly lower (*P* < 0.0001) in pubertal PCOS group than the control and prepubertal PCOS groups (Fig. 1f). The serum LDL and VLDL concentrations were significantly higher (*P* < 0.0001) in pubertal DHEA treated rats than the prepubertal DHEA rats and the control groups (Fig. 1g and h).

**Fig. 2:**
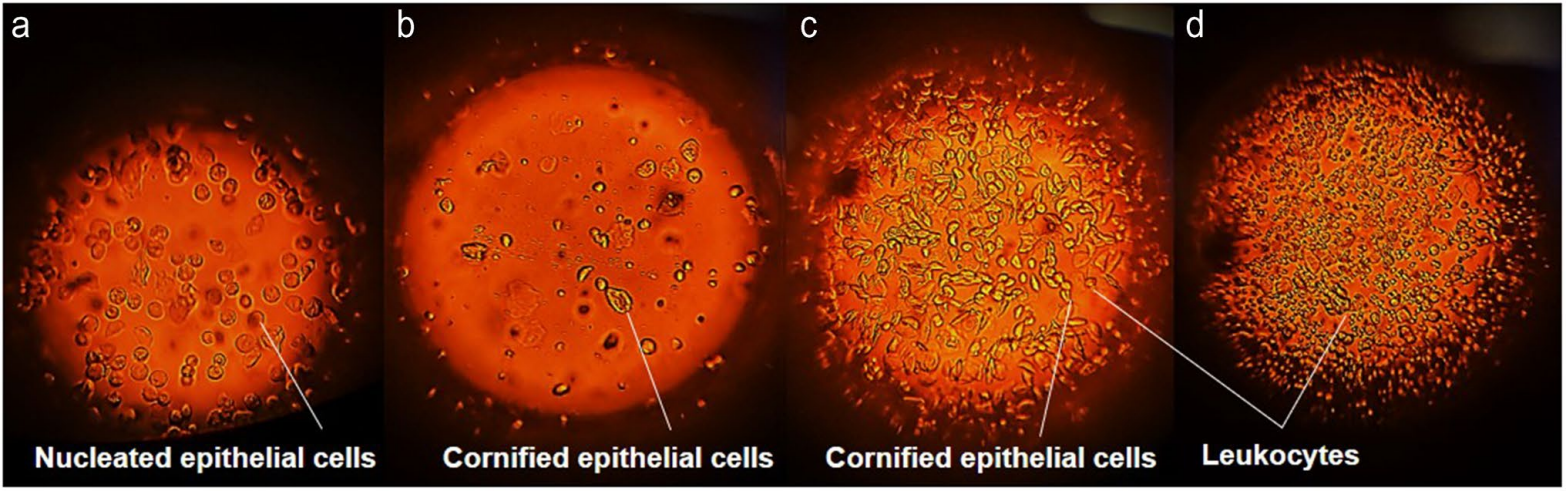
Stages of estrous cycle **a**) proestrus stage, **b**) estrus stage, **c**) metestrus stage, and **d**) diestrus stage as observed under light microscope at 45 × magnification

**Fig. 3:**
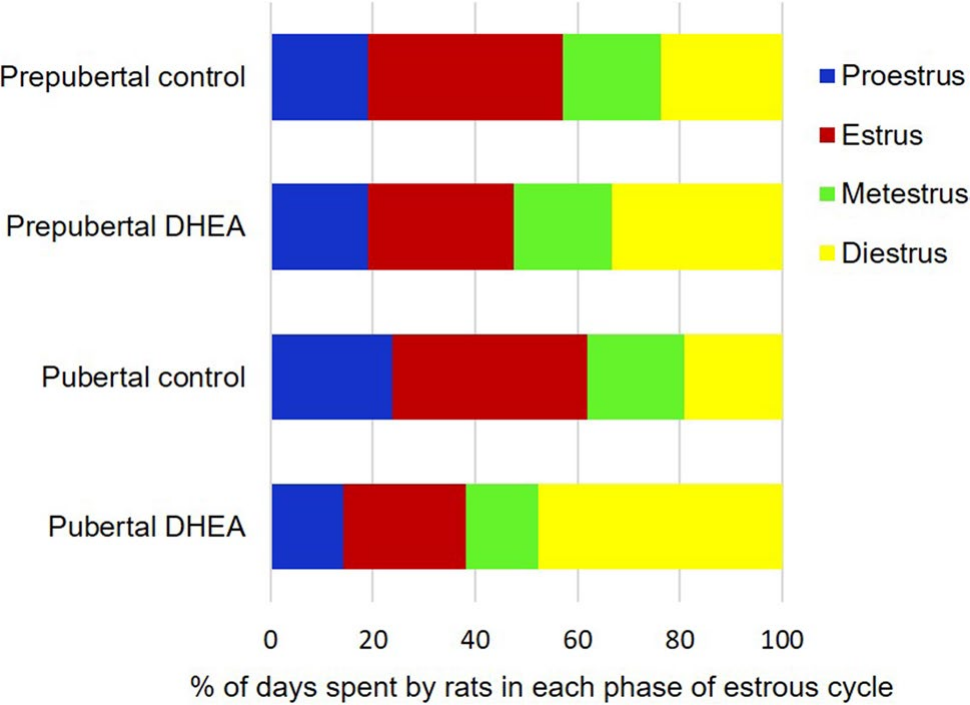
Percentage of days spent by the rats in each stage of the estrous cycle monitored up to 21 days of induction with DHEA

For the rats in control groups, estrous cyclicity was normal in the order proestrus, estrus, metestrus, and diestrus which normally takes five days. The proestrus stage is characterised by the presence of nucleated epithelial cells, estrus stage with cornified epithelial cells, metestrus stage displaying leukocytes and nucleated epithelial cells, and diestrus stage presenting profuse leukocytes represented in Fig. 2. In contrast, the rats in DHEA treated groups showed irregular estrous cycle with prolonged estrus phase and reduced diestrus phase (Fig. 3).

**Fig. 4:**
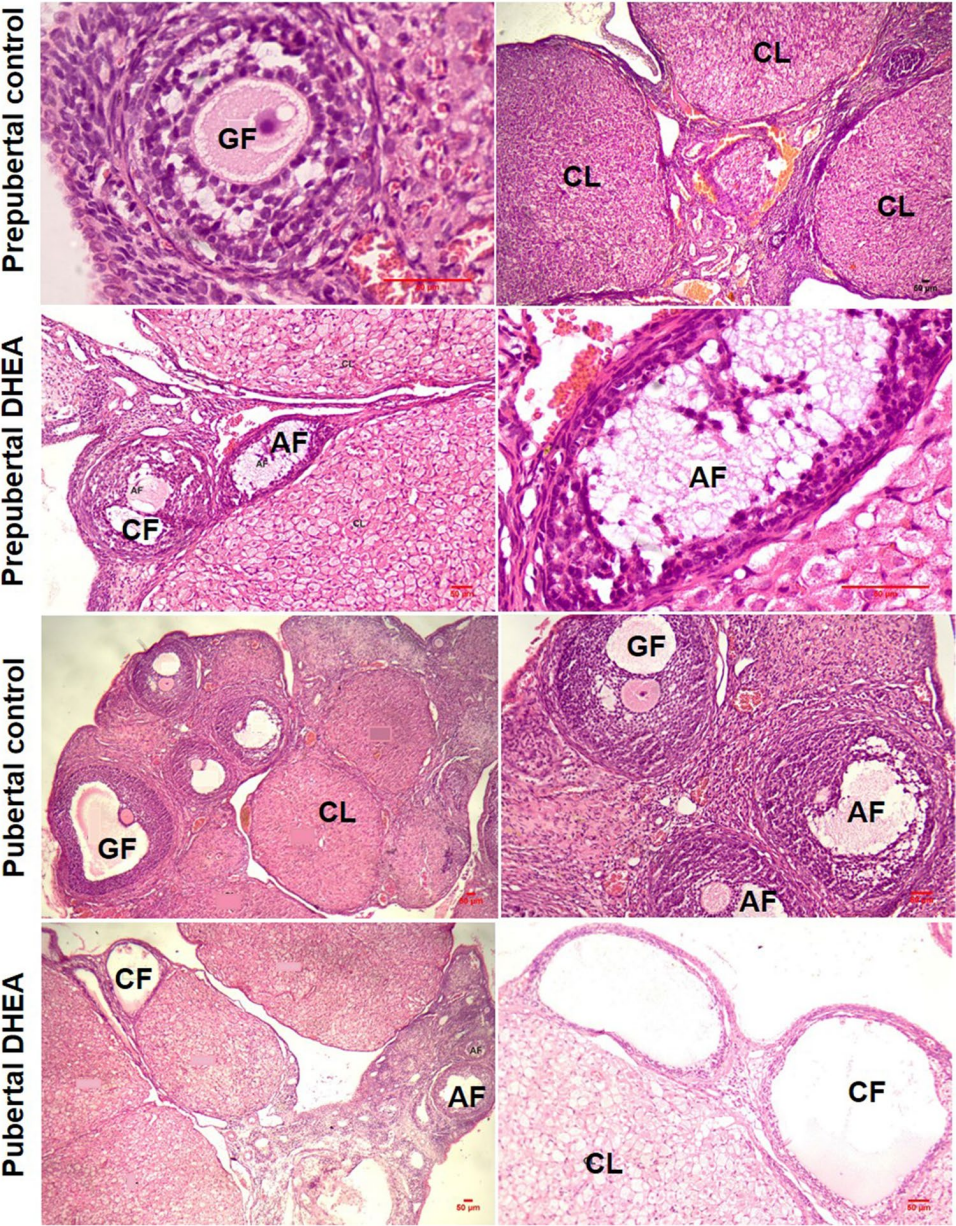
Representative H and E staining of section of ovary tissue excised from pubertal control and DHEA treated rats. The images were taken at a magnification of 100 × and 400 × with 50 µm bars. Control group rats show abundant corpus luteum (CL) and growing follicles (GF). In DHEA treated rats, corpus luteum is reduced in count, while atretic (AF) and cystic follicles (CF) are seen

Polycystic ovaries display upsurge in antral follicles due to androgen excess [21]. As per the histopathology studies, surface epithelium covered ovary tissue was observed in every slide. In both pubertal and prepubertal control group rats, ovary tissue show growing follicles (GF) and corpus luteum in various phases. Degenerating follicles are less in number with well-preserved tissue architecture. Pubertal rats treated with DHEA showed an increased number cystic follicles (CF) compared to prepubertal DHEA treated rats. In PCOS group, numerous degenerating follicles are seen void of GF with decreased count of corpus luteum (CL) (Fig. 4).

### Incorporation of HFD with DHEA and Consecutive Treatment for Improved Metabolic and Reproductive Phenotypes

Though we earlier observed that DHEA treated pubertal rats mimic PCOS-like reproductive trait when compared to prepubertal DHEA model, the lipid profile in accordance to PCOS is not significant. Further improvement to metabolic phenotypes of PCOS was done by including HFD with DHEA. There also exists a need for continual treatment even after induction period of 21 days for the model to sustain reproductive and metabolic features of PCOS. Therefore, the pubertal and prepubertal rats were consecutively treated with DHEA with and without HFD for 21 days (stop dosing), additional 3 weeks on alternative days (continue dosing) and compared to respective control groups.

#### Body Weight

**Fig. 5:**
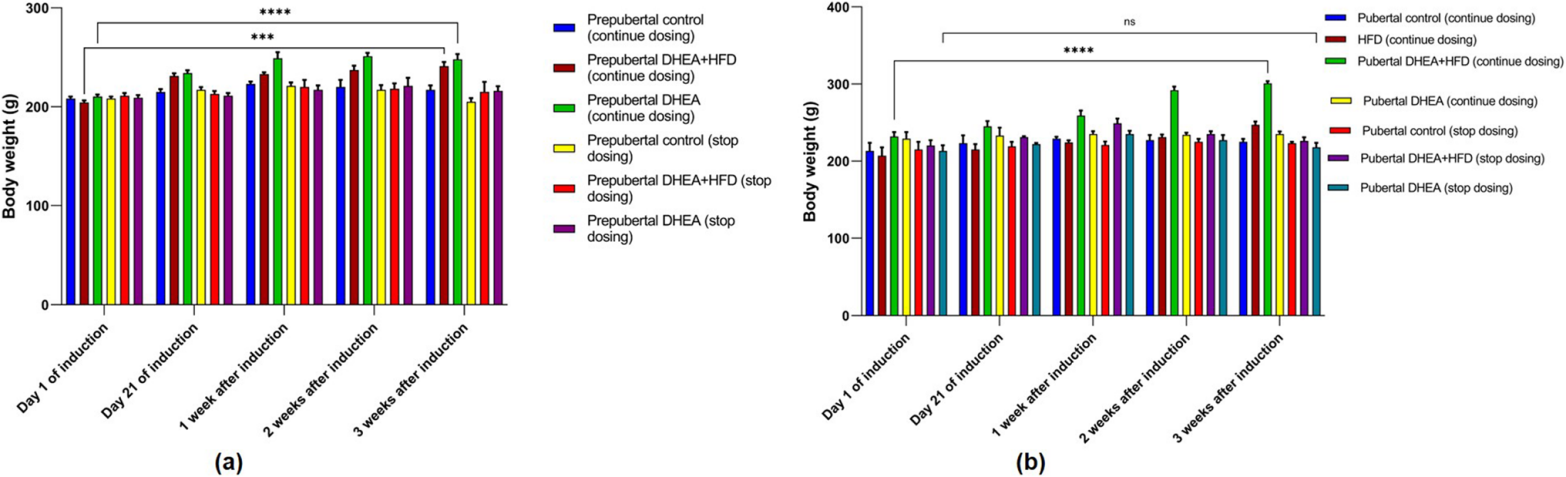
Body weight of (**a**) prepubertal PCOS and control groups (**b**) pubertal PCOS and control groups. The data are presented as mean ± SD and analysed by two-way ANOVA with Tukey’s multiple comparison test

The body weight of rats was monitored weekly for 3 weeks after induction. The prepubertal rats treated with DHEA and DHEA + HFD showed significant increase (*P* < 0.0001) in body weight a week after the induction but remained constant for next 2 weeks. There was no significant difference between the body weight of prepubertal DHEA + HFD and DHEA treated rats (Fig. 5a). The pubertal DHEA + HFD treated rats significantly increased (*P* < 0.0001) the body weight gradually from 21 days to induction to 3 weeks compared to pubertal control, HFD, and pubertal DHEA treated groups (Fig. 5b).

#### Glucose

**Fig. 6:**
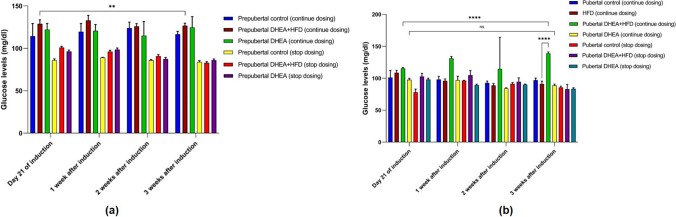
Fasting blood glucose levels in (**a**) prepubertal rats and (**b**) pubertal rats on 21st day of induction to 3 weeks of monitoring. The data are presented as mean ± SD and analysed by two-way ANOVA with Tukey’s multiple comparison test

Prepubertal DHEA + HFD rats in continuous dosing groups showed significant increase (*P* < 0.0001) in blood glucose compared to control from 21 days of induction to 3 weeks. However, improvement after 3 weeks was not observed compared to the glucose level after 21 days of treatment (Fig. 6a). Pubertal HFD fed rats showed a marked increase (*P* < 0.0001) in glucose levels on 21st day of induction compared to control and PCOS continuous and stop dosing groups. In pubertal DHEA + HFD treated rats, significant increase in glucose was seen 3 weeks after induction compared to control and DHEA alone treated rats in control and stop dosing groups. (Fig. 6b).

#### Serum Testosterone Level of the Rats

**Fig. 7:**
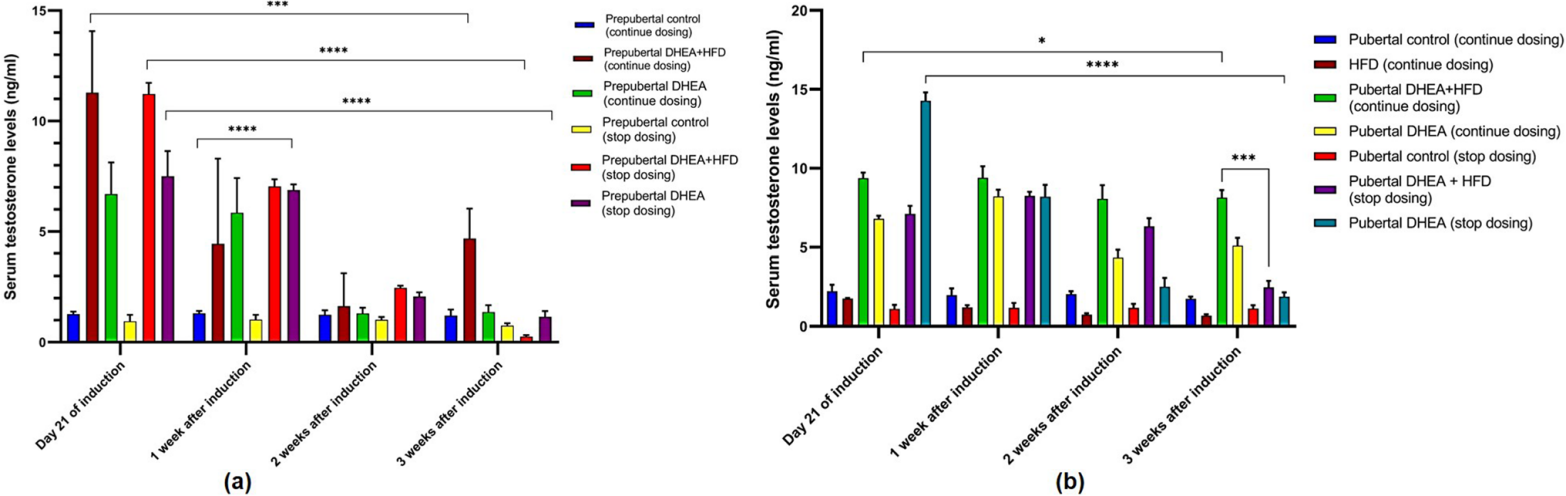
Serum testosterone levels in (**a**) prepubertal rats, (**b**) pubertal rats on 21st day of induction to 3 weeks of monitoring. The data are presented as mean ± SD and analysed by two-way ANOVA with Tukey’s multiple comparison test

Hyperandrogenism is a focal point of PCOS and one of the three criterions of Rotterdam consensus, with underlying associations between elevated testosterone and polycystic ovarian morphology (PCOM) [22]. Although prepubertal rats in DHEA + HFD and DHEA groups showed significant increase (*P* < 0.0001) in the serum testosterone compared to corresponding control groups after 21 days of induction, the concentration remained unchanged for subsequent 3 weeks (Fig. 7a). For the pubertal rats treated with DHEA + HFD, the serum testosterone level was significantly higher (*P* < 0.0001) after 21 days of induction and sustained up to additional three weeks compared to control and DHEA treated group. Contrarily, HFD fed pubertal rats did not have a significant impact on serum testosterone (Fig. 7b).

#### Lipid Profile

**Fig. 8:**
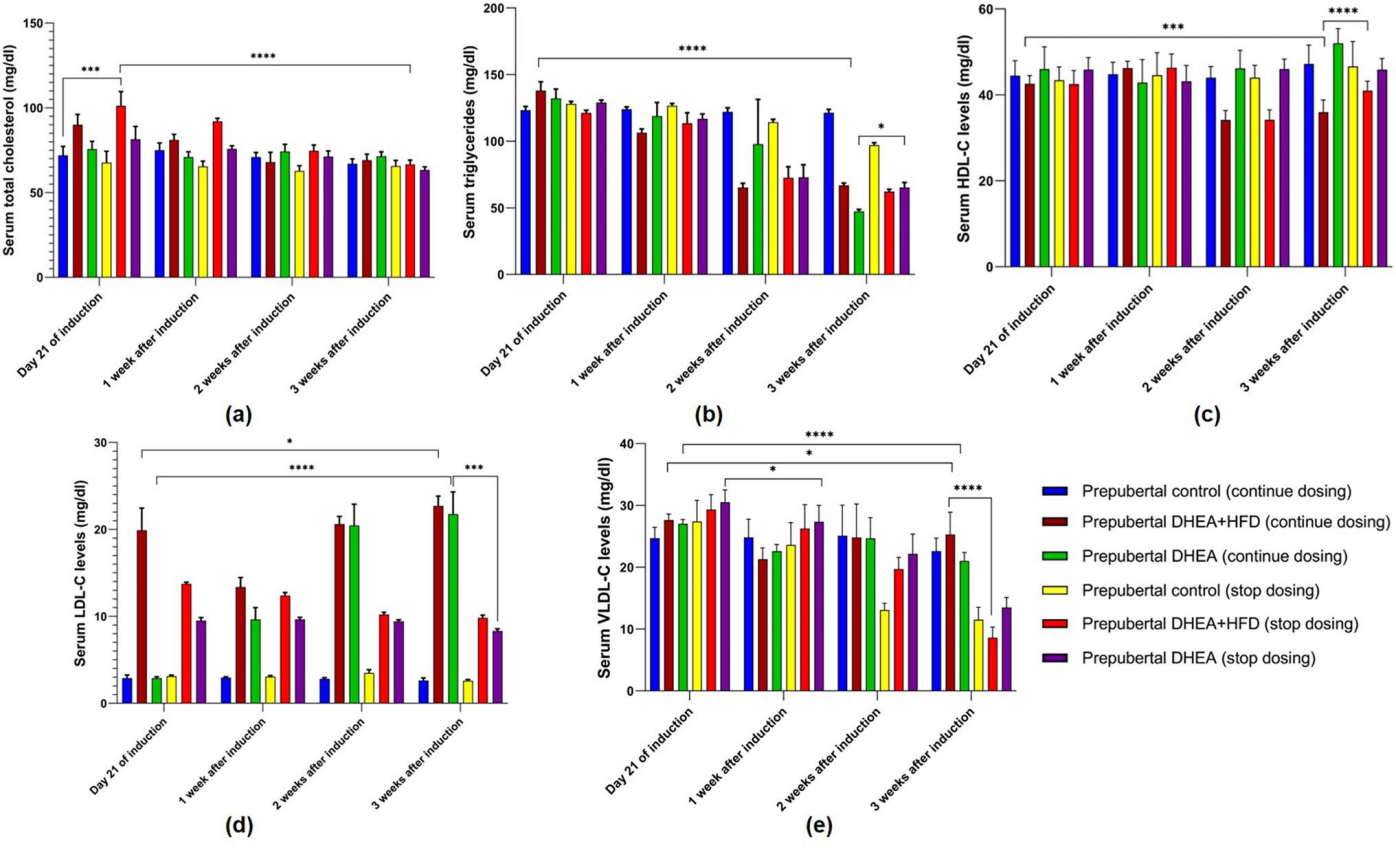
Serum lipid profile **a**) total cholesterol, **b**) triglycerides, **c**) HDL-C, **d**) LDL-C, and **e**) VLDL-C in prepubertal rats on 21st day of induction to 3 weeks of monitoring. The data are presented as mean ± SD and analysed by two-way ANOVA with Tukey’s multiple comparison test

**Fig. 9:**
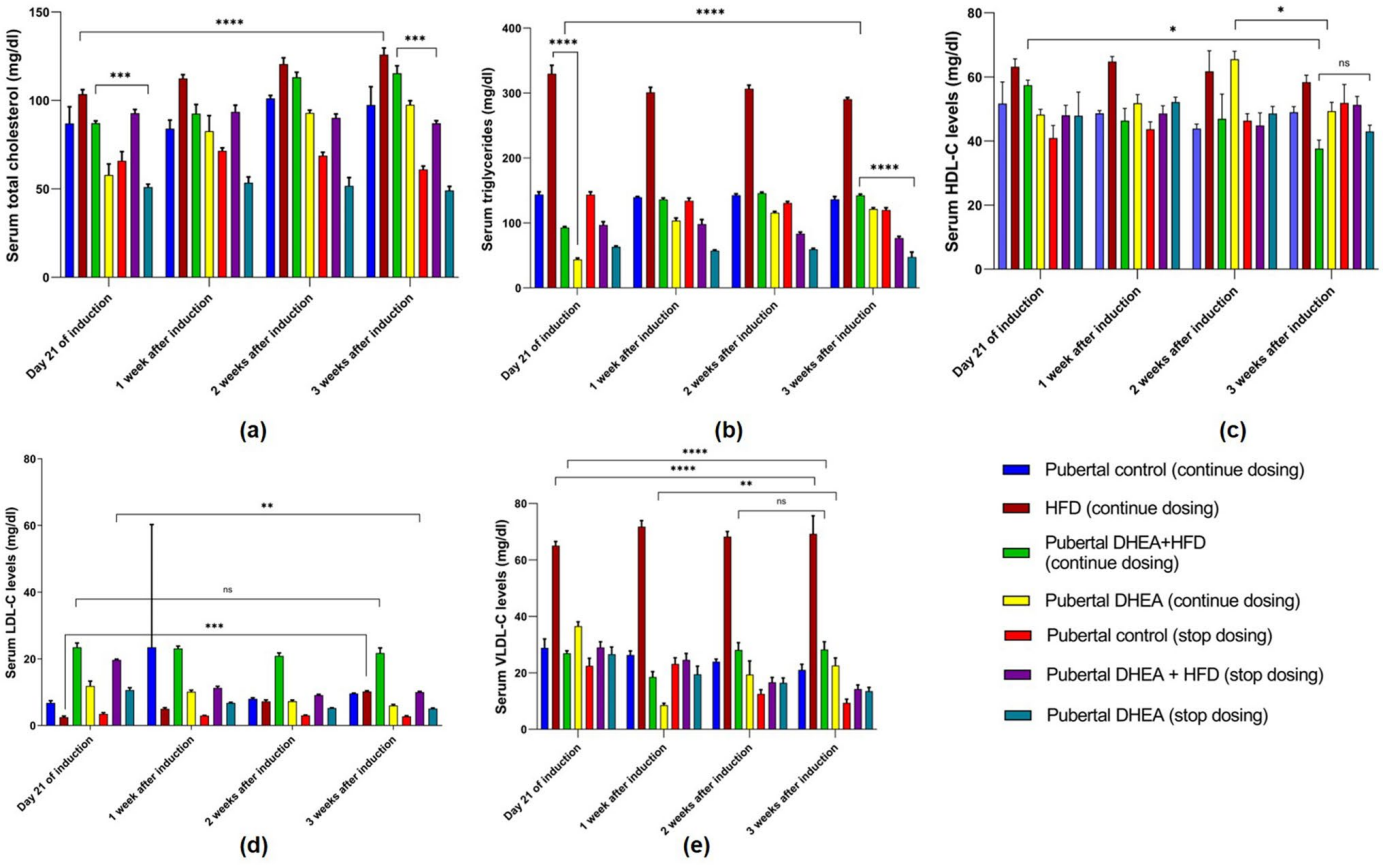
Serum lipid profile **a**) total cholesterol, **b**) triglycerides, **c**) HDL-C, **d**) LDL-C, and **e**) VLDL-C in pubertal rats on 21st day of induction to 3 weeks of monitoring. The data are presented as mean ± SD and analysed by two-way ANOVA with Tukey’s multiple comparison test

Dyslipidaemia is a metabolic disorder often seen in women with PCOS characterised by low HDL level, high LDL-C, TC, and TG levels [23]. In prepubertal rats receiving DHEA and DHEA + HFD, the lipid profiling showed inconsistent results. In continuous dosing groups, there was no marked difference in the TC, LDL-C, and VLDL-C levels compared to control and PCOS stop dosing groups (Fig. 8). The HFD fed group markedly increased TG concentration (*P* < 0.0001) in comparison to continuous dosing DHEA + HFD, DHEA, control, and stop dosing groups. For the pubertal rats in DHEA + HFD continuous dosing group, serum TG, TC, LDL-C, and VLDL-C levels were markedly increased (*P* < 0.0001) after 21 days of induction to 3 weeks than DHEA and control rats in both stop dosing and continuous dosing groups. Pubertal DHEA continuous dosing group showed a significant decrease (*P* < 0.0001) in HDL-C level compared to pubertal DHEA + HFD continuous and stop dosing, HFD, and control groups (Fig. 9).

#### Estrous Cycle

**Fig. 10:**
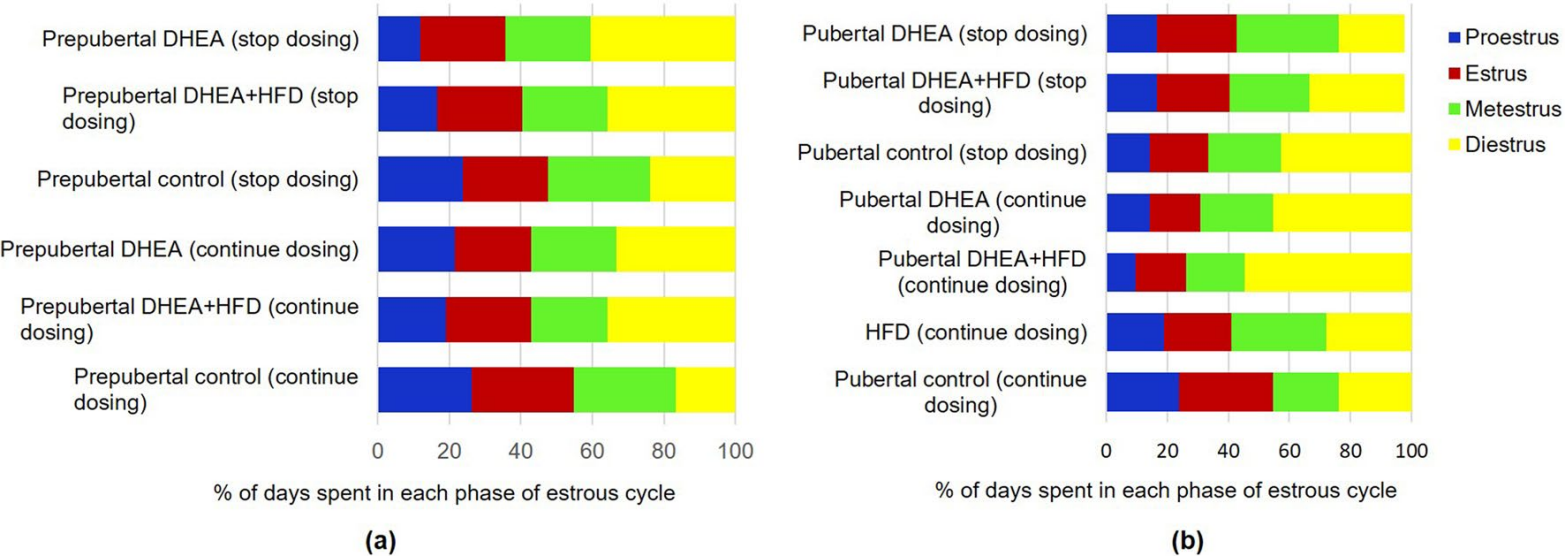
Percentage of days spent by (**a**) prepubertal rats (**b**) pubertal rats of various groups in each stage of the estrous cycle monitored from initial days of PCOS induction to 3 weeks after model development

The estrous cycle was monitored from day 1 of induction to 3 weeks after the model development in all the groups. As illustrated in Fig. 10a and b, prepubertal and pubertal PCOS groups showed irregularity in estrous cycle with prolonged estrus phase and diminished disterus phase, which was dominant in control groups. In stop dosing PCOS groups, the estrous cycle was restored upon withdrawal of DHEA after 21 days.

#### Histopathology results

##### Histopathology Results

**Fig. 11:**
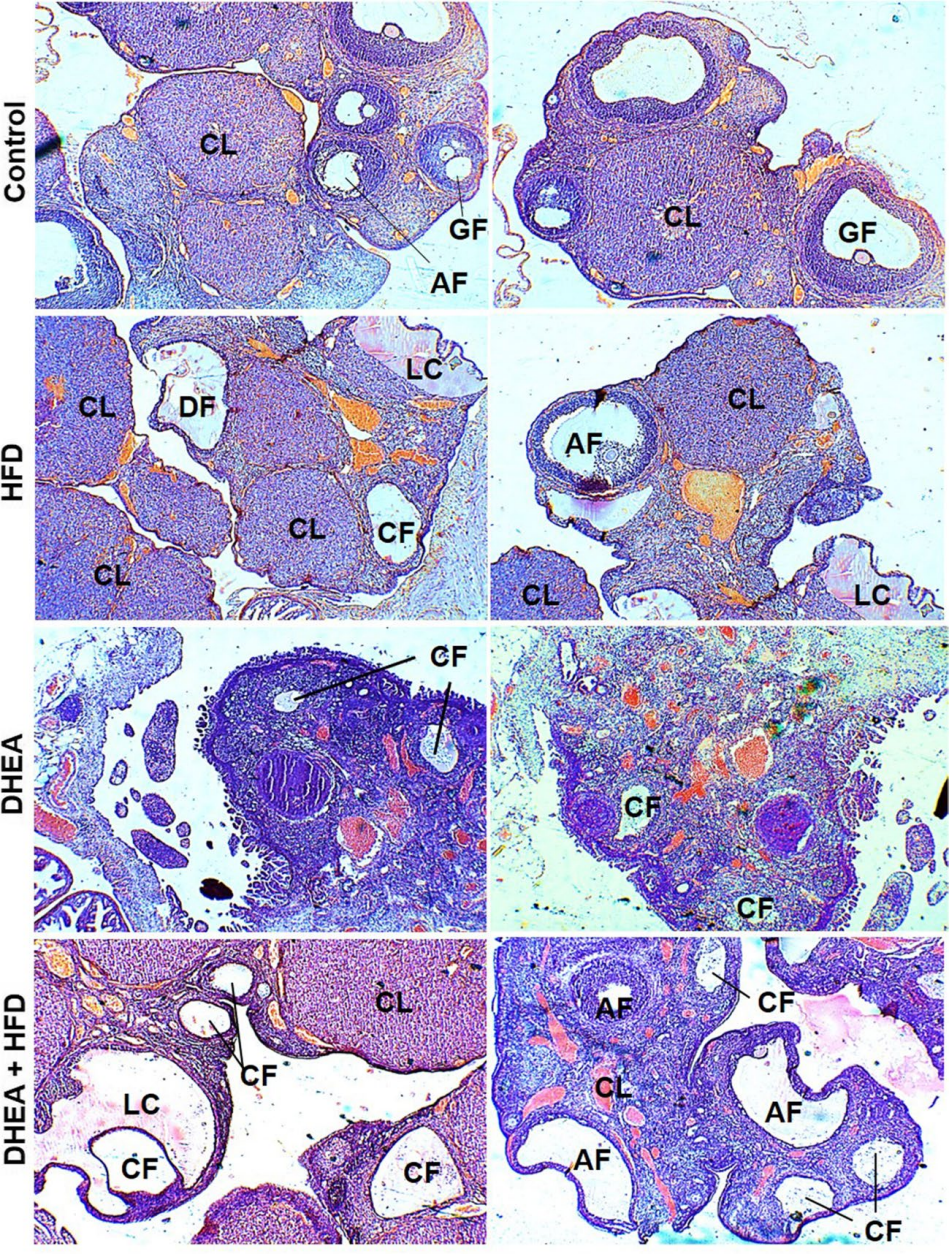
Representative H and E staining of section of ovary tissue excised from control, HFD, DHEA, and DHEA + HFD induced pubertal PCOS rats. The images were taken at a magnification of 10 × and 100 × with scale 1 mm bar. In control, growing follicles (GF), antral follicles (AF), and corpus luteum (CL) at various development stages can be seen while, cystic follicles are not apparent. In HFD group, degenerative follicles (CF) and luteal cysts (LC) are seen with several corpus luteum. Haemorrhagic fluid can be seen inside the corpus luteum. In DHEA group, few cystic follicles (CF) and corpus luteum are seen. In DHEA + HFD group, several cystic follicles (CF), luteal cysts (LC) as well as follicular atresia is evident with decreased count of corpus luteum

**Fig. 12:**
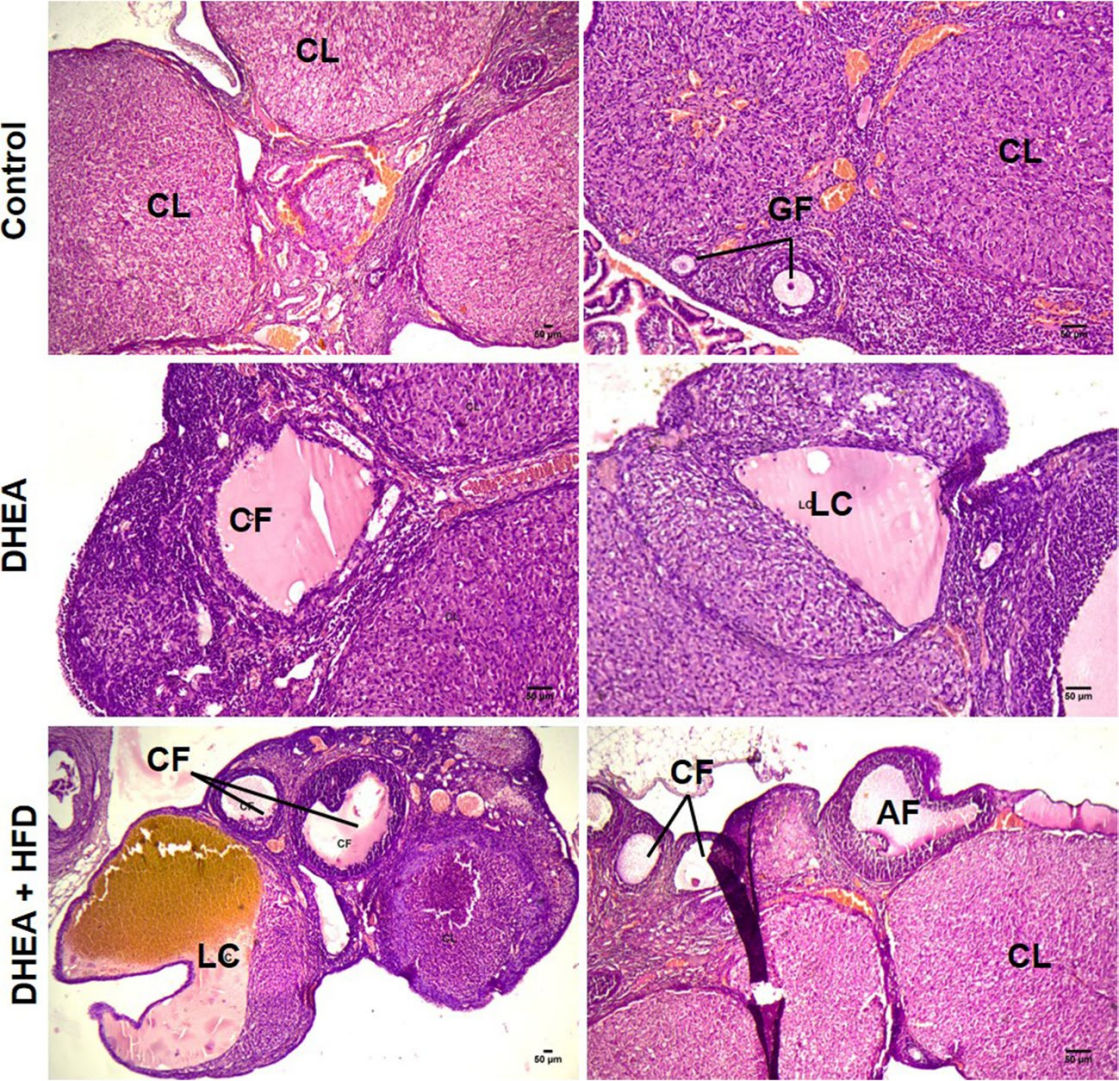
Representative H and E staining of section of ovary tissue excised from control, DHEA, and DHEA + HFD induced prepubertal PCOS rats. The images were taken at a magnification of 40x, 100x, and 400 × with 50 µm bars. In control, growing follicles (GF) and corpus luteum (CL) at various development stages can be seen while, cysts are not evident. In treatment groups, cystic follicles (CF), atretic follicles (AF), and luteal cysts (LC) are seen. Haemorrhagic fluid can be seen inside the corpus luteum

Histopathology studies of the ovaries excised from prepubertal and pubertal control, pubertal HFD, prepubertal and pubertal DHEA and DHEA + HFD treated rats in continuous dosing groups were carried out. Surface epithelium covered ovary tissue was observed in every slide. Follicles in the outer cortex and fibroelastic loose connective tissue with blood and lymphatic vessels in the inner medulla are visible. In both pubertal and prepubertal control group rats, cortex of ovary tissue show growing follicles (GF) in various phases ingrained in the thick stroma. Degenerating follicles were less in number, corpus luteum of different stages and well-preserved tissue architecture is observed. In pubertal rats treated with DHEA, few cystic follicles (CF), numerous degenerating follicles were seen void of GF. Corpus luteum (CL) was reduced in number compared to pubertal control with enlarged, pale, vacuolated, and eosinophilic cells. There was no evidence of antral follicles in control and HFD groups, but increased in DHEA and DHEA + HFD groups. In contrast, cystic follicle count was remarkable in pubertal rats treated with DHEA + HFD. CL was reduced in number with no evidence of GF. Atretic follicles (AF) and CF lined by cuboidal granulosa cells were seen along with atrophied ovary. Follicles were classified as atretic if they displayed > 2 pyknotic nuclei or jagged layer of granulosa cells in a single cross section [20]. Luteal cyst containing eosinophilic fluid was also evident (Fig. 11). As shown in Table 2 and Fig. 12, the prepubertal control rats showed absence of cysts. Whereas, CL and GF of different stages were evident. In prepubertal DHEA treated HFD fed rats, reduction in the number of CL and GF was observed. CF and few luteal cysts enveloped by layers of luteinized granulosa cells with foamy and vacuolated cytoplasm could also be seen and acellular eosinophilic haemorrhagic fluid was seen inside corpus luteum (Tables 1 and 2).

**Table 1:** Histopathology results of the ovary section obtained from pubertal and prepubertal control and PCOS groups of rats after 21 days

Group	Growing follicles in different stages	Atretic/ Degenerating follicles	Corpus luteum	Cystic follicles	Luteal cysts
Control (prepubertal)	5	5	6	0	0
DHEA (pubertal)	0	2	4	3	0
Control (pubertal)	6	2	10	0	0
DHEA (pubertal)	0	1	3	5	1

**Table 2:** Histopathology results of the ovary section obtained from pubertal and prepubertal control and PCOS rats in continuous and stop dosing groups

Group	Growing follicles in different stages	Atretic/ Degenerating follicles	Corpus luteum	Cystic follicles	Luteal cysts
Control (pubertal)	8	2	10	0	0
HFD (pubertal)	0	1	7	3	1
DHEA treated (pubertal)	0	6	5	3	0
DHEA + HFD (pubertal)	0	2	7	6	1
Control (prepubertal)	11	3	14	0	0
DHEA (prepubertal)					
DHEA + HFD (prepubertal)	0	4	5	2	1

## Discussion

Women suffering from PCOS exhibit atypical ovary morphology and irregular menstrual cycle [24]. Hyperandrogenism characterised by elevated serum testosterone and dyslipidemia are the vital PCOS features [23, 25]. Animal models are crucial in explicating pathogenesis of PCOS. An animal model unveiling all the PCOS phenotypes is scant [5]. DHEA induced PCOS rat and mouse model is widely reported [7, 8]. DHEA induces oxidative stress and inflammation resulting in polycystic ovaries as well as hyperandrogenism, anovulation, insulin resistance, and glucose intolerance [10, 11]. The incongruity in reproducing dyslipidemia or hyperlipidaemia confines its reliability [12]. An inevitable drawback of DHEA animal model is the reversal of reproductive and metabolic dysfunction upon withdrawal of treatment. Our study examined the impact of DHEA in establishing PCOS model, focusing on the effects of treatment duration and HFD on reproductive and metabolic phenotypes. The DHEA and DHEA + HFD consecutive treatment groups were compared to stop dosing groups where DHEA with and without treatment was withdrawn after 21 days. The HFD fed PCOS rats displayed drastic increase in lipid levels which could be due to inflation of dylipidemia as a consequence of increased oxidation of fatty acid and fat uptake in liver, decreased efflux of cholesterol and lipogenesis [26]. Any imbalance in hormonal levels disrupts normal functioning of ovaries resulting in cyst formation in the sac of ovaries eventually leading to anovulation or amenorrhea [1]. In our study, PCOS groups exhibited irregular estrous cycle, hyperandrogenism, and polycystic ovaries as reported by Kang et al. (2023) investigating HFD effect and sustainability of the reproductive features in DHEA induced PCOS mice [27]. Consistent with the findings of Kim et al. (2018), our study confirmed that DHEA treated pubertal rats exhibit more pronounced PCOS features such as increased body weight, estrous cycle irregularity, and polycystic ovaries compared to prepubertal rats. However, their study reporting long-term effect of DHEA is inapparent [8]. Our study results also highlight the necessity of including a HFD alongside DHEA to improve the metabolic features of the model. It aligns with research findings by H Zhang et al., 2016 and Kumar et al., 2022 signifying that HFD exacerbates dyslipidemia in rats treated with DHEA, thus producing a more characteristic PCOS animal model replicating human PCOS- specific traits [12, 28]. Zhang et al. (2016) reported that DHEA + HFD treatment markedly increased serum TG, TC, LDL, VLDL levels, and decreased HDL levels, similar to our findings [28]. Our histopathological study findings revealed that DHEA and DHEA + HFD treatment led to substantial changes in ovary inclusive of increased cystic follicles, luteal cysts, degenerative follicles, and reduced corpus luteum, predominantly in pubertal rats compared to prepubertal rats. These results align with data reported by Wang et al., 2022, Wu et al., 2023, and Kumar et al., 2022 demonstrating similar morphological alterations of ovaries treated with DHEA [7, 12, 15].

## Conclusion

To conclude, our study emphasizes the effectiveness of the DHEA-induced PCOS rat model, particularly when co-administered with HFD and prolonged treatment period, to reproduce the PCOS-specific metabolic and reproductive traits more adeptly. Accordingly, consecutive dosing of rats with DHEA concomitantly with high fat diet is essential to sustain PCOS-specific phenotypes desirable for therapeutic interventions, especially evaluation of formulations employing varied strategies to treat PCOS. Future research should focus to advance this model and identify additional factors inducing metabolic and reproductive dysfunctions in PCOS.

## Data Availability

The data supporting this study's findings are available from the corresponding author upon request.
